# Supplementary material for: Robust Emax model fitting: Addressing nonignorable missing binary outcome in dose–response analysis
Source: Stat Methods Med Res. 2025 Dec 29;35(2):255–67. doi: 10.1177/09622802251403356 (PMC13036265; doi:10.1177/09622802251403356)
Supplement: sj-pdf-1-smm-10.1177_09622802251403356 - Supplemental material for Robust Emax model fitting: Addressing nonignorable missing binary outcome in dose–response analysis [file sj-pdf-1-smm-10.1177_09622802251403356.pdf]

Supplementary Material to Robust Emax Model Fitting: Addressing Nonignorable Missing Binary Data in Dose Response Analysis

September 17, 2025

1 M-step of Weighted EM Procedure of IL and FIL

In this section, we present the detailed derivation of the M-step of both IL and FIL methods.

1.1 M-step of Weighted EM Procedure of IL

The maximization of $l(\boldsymbol{\alpha}|z_i, r_i)$ is straightforward, which can be done iteratively by the Newton-Raphson method with the following updating equation:

$$\boldsymbol{\alpha}^{(s+1,t)} = \boldsymbol{\alpha}^{(s,t)} + I(\boldsymbol{\alpha}^{(s,t)})^{-1} U(\boldsymbol{\alpha}^{(s,t)}) \tag{1}$$

where $\boldsymbol{\alpha}^{(s,t)}$ is the estimate of $\boldsymbol{\alpha}$ at s-th iteration of the Newton-Raphson procedure within t-th iteration of the EM algorithm, $I(\boldsymbol{\alpha}) = Z^T V(\boldsymbol{\alpha}) Z$ is the information matrix at $\boldsymbol{\alpha}$ with

$V(\boldsymbol{\alpha}) = \text{diag}(w_{iy_i} p_i(1 - p_i))$, and $U(\boldsymbol{\alpha})$ is the score function defined as in the following:

$$U(\boldsymbol{\alpha}) = \sum_{i=1}^{n} \sum_{y_i=0}^{1} w_{iy_i} z_i (r_i - p_i). \quad (2)$$

The entire procedure can be implemented using standard software packages that fit logistic regression with specified weight options. Next, for the M-step of $l(\boldsymbol{\theta}|Dose_i, y_i)$, consider the Newton-Raphson method to find the maximizer. After some algebra, the score function reduces to

$$U(\boldsymbol{\theta}) = \sum_{i=1}^{n} \sum_{y_i=0}^{1} w_{iy_i} (y_i - \pi_i) \nabla \eta(Dose_i, \boldsymbol{\theta}) \quad (3)$$

where $\eta(Dose_i, \boldsymbol{\theta}) = E_0 + E_{max} \times Dose_i/(ED_{50} + Dose_i)$, ∇ is the differential operator with respect to $\boldsymbol{\theta}$, and

$$\nabla \eta(Dose_i, \boldsymbol{\theta}) = \left(1, \frac{Dose_i}{Dose_i + ED_{50}}, -\frac{Dose_i \times E_{max}}{(Dose_i + ED_{50})^2}\right)^T.$$

The Hessian matrix is:

$$H(\boldsymbol{\theta}) = \sum_{i=1}^{n} \sum_{y_i=0}^{1} w_{iy_i} \left((\pi_i - 1)\pi_i \nabla \eta(Dose_i, \boldsymbol{\theta})^\top \nabla \eta(Dose_i, \boldsymbol{\theta}) - A_i(\boldsymbol{\theta})\right) \quad (4)$$

where

$$A_i(\boldsymbol{\theta}) = \begin{pmatrix} 0 & 0 & 0 \\ 0 & 0 & \frac{(y_i - \pi_i)Dose_i}{(ED_{50} + Dose_i)^2} \\ 0 & \frac{(y_i - \pi_i)Dose_i}{(ED_{50} + Dose_i)^2} & -\frac{2(y_i - \pi_i)Dose_i \times E_{max}}{(ED_{50} + Dose_i)^3} \end{pmatrix}.$$

The observed information matrix can be obtained by taking the negative value of the Hessian matrix, and the updating equation of $\boldsymbol{\theta}$ is similar as (1) of $\boldsymbol{\alpha}$. It is pertinent to point out that $A_i(\boldsymbol{\theta})$ is the additional term due to non-linear model setting compared to the Hessian matrix of logistic regression of i-th observation when weight $w_{iy_i} = 1$.

1.2 M-step of Weighted EM Procedure of FIL

For maximizing $l^*(\boldsymbol{\alpha}|r_i, z_i)$, the modified score function $U^*(\boldsymbol{\alpha})$ takes the form as

$$U^*(\boldsymbol{\alpha}) = \sum_{i=1}^{n} \sum_{y_i=0}^{1} w_{iy_i} z_i [y_i - p_i + h_i(1/2 - p_i)], \tag{5}$$

where h_i is the i-th diagonal elements of the hat matrix $V^{1/2}Z(Z^\top VZ)^{-1}Z^\top V^{1/2}$. For maximizing $l^*(\boldsymbol{\theta}|y_i, Dose_i)$, the corresponding score function $U^*(\boldsymbol{\theta})$ is formulated as:

$$U^*(\boldsymbol{\theta}) = \sum_{i=1}^{n} \sum_{y_i=0}^{1} w_{iy_i}(y_i - \pi_i)\nabla\eta(Dose_i, \boldsymbol{\theta}) + \mathrm{tr}\left(I(\boldsymbol{\theta})^{-1}\frac{\partial I}{\partial \boldsymbol{\theta}}\right). \tag{6}$$

2 Computational considerations of the algorithm

We fit the binary Emax model and the logistic model for missingness by maximizing the Jeffreys prior-penalized observed-data log-likelihood using Newton/Fisher scoring with backtracking line search. We reparameterize $ED_{50} = \exp(\log ED_{50})$ to enforce positivity.

Initialization. We obtain starting values by (i) fitting the Emax model with complete cases to initialize $\boldsymbol{\theta} = (E_0, ED_{50}, E_{max})^\top$; (ii) fitting a logistic regression for R on $(X, Dose)$ to initialize $\boldsymbol{\alpha}$ and setting the MNAR coefficent $\alpha_{(p+2)}$ to 0. We then perform a small number of random or grid perturbations around these starts and retain the best objective value.

Convergence checks. Iterations stop when the relative change in the (penalized) log-likelihood is below 10^{-5}, or after a maximum of 200 iterations. We monitor the conditioning of the observed information; if it is ill-conditioned, we switch to a trust-region/quasi-Newton update. Final fits are required to have a positive-definite observed information (Hessian).

Sensitivity to initial values. In our experience, differences across initial values are negligible when the MNAR signal is identifiable; larger variability of estimation due to choice of initial value is a diagnostic of weak identification of α_Y rather than algorithmic pathology.

Computational complexity. For iteration, gradients and observed information are sums of per-subject contributions ($O(n)$), and Hessian inversion requires $O(p^3)$ of runtime for p parameters. With p fixed and small for the models, runtime scales approximately linearly in n.

3 Addtional simulation result

3.1 Operating missingness and dose correlation

This Section provides outputs when we generated the simulated datasets with higher correlation between missingness and dose. Keeping other settings the same as Section 3, we change true α to be:

$$\alpha = (0.5, -3, 0, 3, 1).$$

The setting leads to severe separation in the placebo treatment arm, while the MNAR is comparably small compared to MAR due to dose assignment. We present simulation results as Table S1. The seemingly strong MSE performance for MI in Table S1 arises because MSE blends bias and variance; in this particular configuration, the MNAR effect was moderate

while there exists a strong missingness predictor, dosage. The imputation model included this strong observed predictor, so MI's variance was especially small and outweighed its (nonzero) bias—yielding a low MSE. Still FIL has the lowest bias estimation and comparably lowest RMSE, suggesting stable inference performance.

3.2 Operating missingness rate

This Section provides outputs when we generated the simulated datasets with different missing rates. In specific, we keep other settings the same as Section 3, and change true α_1 to be 1, 3, 6, and 8 to produce 10%, 15%, 25%, and 30% missing rate. We present simulation results as Table S2. the results are similar to those in Section 3. FIL outperforms IL, and both methods provide better estimates than CC, NRI, and MI. Additionally, FIL consistently achieves the lowest mean estimated standard errors, with coverage probabilities close to the desired 95% nominal level.

3.3 Operating level of nonignorable missingness

This Section provides outputs when we generated the simulated datasets with different levels of nonignorable missingness. In specific, we keep other settings the same as Section 3, and change true α_4 to be 0,1,3, and 5. Note that $\alpha_4 = 0$ suggests the missingness is MAR. We present simulation results as Table S3. It can been seen that when $\alpha_4 = 0$, MI has better performance in term of RMSE, while FIL still has the lowest MBE due to potential separation. As the level of nonignorable missingness increases, the performance of MI becomes worse, with FIL and IL outperforming the others.

Sample Size(N)	Parameter	Type	Estimate	MBE	RMSE	Est.SE	CP	Est.Length
250	$\log(ED_{50})$	CC	1.900	**-0.115**	**0.460**	0.568	0.990	2.225
		NRI	1.548	-0.467	0.594	**0.430**	0.890	**1.684**
		MI	2.271	0.256	0.658	0.486	0.967	1.905
		IL	2.998	0.983	2.116	0.703	0.687	2.758
		FIL	2.219	0.204	0.680	0.568	**0.960**	2.227
	E_{max}	CC	4.057	**0.473**	0.702	0.915	1.000	3.585
		NRI	5.105	1.522	1.592	0.928	0.813	3.636
		MI	3.632	0.048	0.536	**0.581**	**0.967**	**2.277**
		IL	3.853	0.270	1.260	1.043	0.807	4.087
		FIL	3.594	**0.011**	0.601	0.782	0.957	3.065
	E_0	CC	-2.623	0.532	0.614	0.925	1.000	3.628
		NRI	-3.706	-1.508	1.559	0.920	0.997	3.606
		MI	-2.104	0.124	**0.446**	0.611	0.930	**2.004**
		IL	-1.921	0.276	2.159	0.703	0.657	2.613
		FIL	-2.117	**0.080**	0.609	**0.568**	**0.957**	2.920
350	$\log(ED_{50})$	CC	1.783	-0.198	0.443	0.476	0.970	1.865
		NRI	1.476	-0.523	0.618	**0.367**	0.742	**1.438**
		MI	2.048	0.233	**0.354**	0.493	0.990	1.541
		IL	2.822	0.762	2.018	0.590	0.840	2.312
		FIL	2.092	**0.116**	0.524	0.497	**0.952**	1.949
	E_{max}	CC	4.283	0.626	0.836	0.875	0.998	3.432
		NRI	5.333	1.690	1.768	0.887	0.498	3.478
		MI	3.833	0.249	**0.589**	**0.522**	**0.950**	**2.047**
		IL	3.859	0.200	1.369	0.912	0.684	3.575
		FIL	3.681	**0.132**	0.668	0.727	0.954	2.848
	E_0	CC	-2.885	-0.607	0.794	0.888	0.998	3.480
		NRI	-3.957	-1.697	1.760	0.885	0.330	3.469
		MI	-2.395	-0.197	**0.523**	**0.480**	0.942	**1.881**
		IL	-2.054	0.174	1.463	0.646	0.484	2.534
		FIL	-2.250	**-0.035**	0.643	0.722	**0.943**	2.830
450	$\log(ED_{50})$	CC	1.786	-0.229	0.432	0.419	0.960	1.644
		NRI	1.477	-0.538	0.606	0.325	0.663	1.273
		MI	2.143	0.153	**0.346**	**0.341**	0.960	**1.339**
		IL	2.564	0.549	1.606	0.508	0.553	1.991
		FIL	2.168	**0.128**	0.597	0.457	**0.943**	1.793
	E_{max}	CC	4.286	0.702	0.897	0.783	1.000	3.071
		NRI	5.332	3.135	1.322	3.371	0.230	3.111
		MI	3.867	0.284	**0.593**	**0.462**	0.913	**1.810**
		IL	3.880	0.297	1.321	0.758	0.573	2.972
		FIL	3.627	**0.043**	0.670	0.639	**0.953**	2.503
	E_0	CC	-2.885	-0.688	0.879	0.793	0.997	3.108
		NRI	-3.953	-1.756	1.835	0.792	0.127	3.103
		MI	-2.419	-0.222	**0.543**	**0.425**	0.933	**1.664**
		IL	-2.196	**0.021**	1.416	0.595	0.727	2.333
		FIL	-2.196	**0.021**	0.708	0.628	**0.956**	2.461

Table S1: Estimates, absolute bias, root mean squared error, estimated standard errors, coverage probabilities and 95% Wald confidence intervals based on 1000 simulations with missing rate≈15% with uneven missing pattern.

Missing rate (%)	Parameter	Type	Estimate	MBE	RMSE	Est.SE	CP	Est.Length
10	$\log(ED_{50})$	CC	2.014	**-0.001**	0.426	0.418	0.956	1.637
		NRI	2.275	0.260	0.509	0.413	0.898	1.619
		MI	2.076	0.061	0.423	0.394	0.944	1.543
		IL	1.979	-0.036	0.438	0.422	**0.955**	1.656
		FIL	2.009	-0.006	**0.394**	**0.393**	0.956	**1.540**
	E_{max}	CC	3.742	0.158	0.562	0.509	0.963	1.995
		NRI	3.833	0.249	0.566	0.489	**0.952**	1.918
		MI	3.699	0.116	0.513	**0.467**	**0.948**	**1.829**
		IL	3.678	0.094	0.551	0.506	0.958	1.984
		FIL	3.639	**0.056**	**0.504**	0.489	0.961	1.916
	E_0	CC	-2.346	-0.149	0.531	0.469	0.976	1.837
		NRI	-2.482	-0.285	0.572	0.458	0.974	1.794
		MI	-2.277	-0.080	0.465	**0.419**	**0.950**	**1.641**
		IL	-2.282	-0.085	0.518	0.465	0.957	1.823
		FIL	-2.255	**-0.058**	**0.464**	0.445	0.964	1.746
15	$\log(ED_{50})$	CC	2.087	0.072	0.448	0.436	0.962	1.708
		NRI	2.514	0.499	0.673	0.423	0.783	1.657
		MI	2.162	0.147	0.447	**0.394**	0.925	**1.543**
		IL	2.005	**-0.010**	0.459	0.449	0.967	1.759
		FIL	2.041	0.026	**0.412**	0.414	**0.959**	1.624
	E_{max}	CC	3.863	0.280	0.649	0.553	0.962	2.169
		NRI	4.106	0.523	0.743	0.519	0.896	2.034
		MI	3.816	0.232	0.603	**0.485**	0.937	**1.901**
		IL	3.712	0.128	0.626	0.550	0.944	2.155
		FIL	3.670	**0.086**	**0.561**	0.526	**0.949**	2.060
	E_0	CC	-2.436	-0.238	0.603	0.520	0.984	2.037
		NRI	-2.653	-0.455	0.696	0.493	0.958	1.933
		MI	-2.365	-0.168	0.544	**0.440**	0.957	**1.725**
		IL	-2.295	-0.098	0.583	0.514	0.942	2.017
		FIL	-2.265	**-0.067**	**0.509**	0.487	**0.956**	1.909
25	$\log(ED_{50})$	CC	2.215	0.200	0.527	0.477	0.939	1.871
		NRI	2.945	0.930	1.083	0.451	0.480	1.770
		MI	2.288	0.273	0.508	**0.384**	0.872	**1.507**
		IL	2.076	0.061	0.914	0.523	0.956	2.049
		FIL	2.064	**0.049**	**0.475**	0.465	**0.950**	1.821
	E_{max}	CC	4.164	0.581	0.871	0.678	0.961	2.656
		NRI	4.696	1.112	1.253	0.599	0.546	2.348
		MI	4.161	0.578	0.832	**0.544**	0.862	**2.131**
		IL	3.719	0.135	0.819	0.695	0.918	2.725
		FIL	3.674	**0.091**	**0.640**	0.620	**0.945**	2.431
	E_0	CC	-2.706	-0.509	0.810	0.662	0.993	2.596
		NRI	-3.060	-0.863	1.023	0.563	0.790	2.205
		MI	-2.658	-0.460	0.741	**0.507**	0.940	**1.988**
		IL	-2.247	**-0.050**	0.764	0.635	0.893	2.487
		FIL	-2.262	-0.065	**0.595**	0.592	**0.941**	2.321
30	$\log(ED_{50})$	CC	2.244	0.229	0.563	0.505	0.921	1.980
		NRI	3.339	1.324	1.476	0.569	0.365	2.231
		MI	2.392	0.377	0.610	**0.418**	0.830	**1.640**
		IL	2.099	0.085	1.071	0.533	0.961	2.088
		FIL	2.038	**0.023**	**0.476**	0.475	**0.958**	1.860
	E_{max}	CC	4.123	0.540	0.809	0.681	0.979	2.670
		NRI	4.438	0.854	1.039	0.640	0.812	2.507
		MI	4.032	0.449	0.779	**0.545**	0.892	**2.136**
		IL	3.740	0.156	0.800	0.785	0.937	3.077
		FIL	3.673	**0.089**	**0.600**	0.626	**0.956**	2.455
	E_0	CC	-2.705	-0.508	0.791	0.665	0.996	2.607
		NRI	-2.843	-0.646	0.826	0.528	0.921	2.072
		MI	-2.604	-0.406	0.716	**0.499**	0.942	**1.955**
		IL	-2.263	-0.066	0.759	0.635	0.899	2.490
		FIL	-2.230	**-0.033**	**0.577**	0.597	**0.950**	2.340

Table S2: Estimates, mean bias error, root mean squared error, estimated standard errors, coverage probabilities, and 95% Wald confidence intervals based on 1000 simulations with sample size n=350.

α_{p+2}	Parameter	Type	Estimate	MBE	RMSE	Est.SE	CP	Est.Length
0	$\log(ED_{50})$	CC	1.964	-0.051	0.453	0.421	0.941	1.651
		NRI	2.283	0.268	0.493	0.394	0.872	1.543
		MI	2.150	0.135	**0.405**	0.395	0.963	1.547
		IL	1.969	-0.046	0.464	0.419	0.941	1.644
		FIL	2.002	**-0.013**	0.419	**0.391**	**0.952**	**1.533**
	E_{max}	CC	3.783	0.206	0.622	0.543	0.980	2.128
		NRI	4.034	0.451	0.706	0.516	0.969	2.024
		MI	3.549	**-0.134**	**0.496**	**0.448**	**0.940**	**1.755**
		IL	3.790	0.156	0.615	0.542	0.974	2.123
		FIL	3.725	0.142	0.540	0.515	0.965	2.019
	E_0	CC	-2.359	-0.162	0.628	0.502	0.968	1.967
		NRI	-2.604	-0.407	0.706	0.484	0.940	1.899
		MI	-2.095	0.132	**0.479**	**0.390**	0.850	**1.529**
		IL	-2.365	-0.168	0.623	0.500	0.962	1.961
		FIL	-2.311	**-0.114**	0.544	0.471	**0.958**	1.846
1	$\log(ED_{50})$	CC	2.002	-0.113	0.439	0.423	0.965	1.657
		NRI	2.407	0.392	0.595	0.412	0.823	1.617
		MI	2.213	0.198	0.429	**0.391**	0.930	**1.533**
		IL	1.936	-0.079	0.479	0.437	0.953	1.712
		FIL	1.976	**-0.039**	**0.424**	0.403	**0.950**	1.582
	E_{max}	CC	3.937	0.354	0.629	0.565	0.993	2.213
		NRI	4.144	0.560	0.722	0.526	0.912	2.061
		MI	3.737	0.154	**0.478**	**0.465**	0.935	**1.824**
		IL	3.778	0.195	0.588	0.559	0.960	2.192
		FIL	3.735	**0.152**	0.514	0.534	**0.957**	2.093
	E_0	CC	-2.518	-0.321	0.603	0.533	0.987	2.088
		NRI	-2.730	-0.533	0.714	0.506	0.952	1.985
		MI	-2.285	-0.168	**0.419**	**0.417**	0.942	**1.636**
		IL	-2.361	-0.088	0.566	0.525	0.960	2.057
		FIL	-2.328	**-0.073**	0.485	0.496	**0.956**	1.945
3	$\log(ED_{50})$	CC	2.274	0.259	0.552	0.456	0.921	1.786
		NRI	2.975	0.960	1.102	0.469	0.504	1.839
		MI	2.540	0.525	0.680	**0.413**	0.772	**1.618**
		IL	1.948	-0.067	0.528	0.480	0.960	1.881
		FIL	1.996	**-0.019**	**0.461**	0.435	**0.954**	1.706
	E_{max}	CC	4.294	0.711	0.941	0.648	0.892	2.541
		NRI	4.489	0.906	1.055	0.577	0.732	2.260
		MI	4.171	0.587	0.802	**0.534**	0.813	**2.092**
		IL	3.721	0.138	0.637	0.607	0.961	2.379
		FIL	3.704	**0.121**	**0.568**	0.578	**0.960**	2.265
	E_0	CC	-2.875	-0.678	0.915	0.632	1.000	2.478
		NRI	-2.958	-0.761	0.936	0.546	0.870	2.140
		MI	-2.716	-0.519	0.722	**0.504**	0.980	**1.975**
		IL	-2.344	-0.147	0.606	0.579	0.940	2.269
		FIL	-2.337	**-0.140**	**0.532**	0.547	**0.958**	2.145
5	$\log(ED_{50})$	CC	2.790	0.775	0.913	0.504	0.689	1.975
		Impu_0	3.929	1.914	1.988	0.527	0.048	2.064
		MI	3.125	1.110	1.215	0.457	0.366	1.792
		IL	2.040	0.054	0.488	0.471	0.958	1.845
		FIL	2.068	**0.025**	**0.441**	**0.437**	**0.955**	**1.712**
	E_{max}	CC	4.918	1.334	1.420	0.723	0.617	2.836
		Impu_0	5.511	1.927	2.063	0.779	0.205	3.053
		MI	4.882	1.298	1.379	0.616	0.404	2.413
		IL	3.781	0.197	0.565	0.611	0.970	2.396
		FIL	3.760	**0.176**	**0.503**	**0.587**	**0.963**	**2.300**
	E_0	CC	-3.253	-1.055	1.183	0.729	0.968	2.859
		Impu_0	-3.150	-0.953	1.037	0.511	0.612	2.001
		MI	-3.128	-0.931	1.032	0.571	0.791	2.237
		IL	-2.301	-0.104	0.553	0.587	0.960	2.301
		FIL	-2.296	**-0.098**	**0.479**	**0.559**	**0.958**	**2.193**

Table S3: Estimates, mean bias error, root mean squared error, estimated standard errors, coverage probabilities, and 95% Wald confidence intervals based on 1000 simulations with sample size n=350 across different α_{p+2} settings.

3.4 Operating sample sizes

This Section provides outputs when we generated the simulated datasets with additional sample size settings. In specific, we keep other settings the same as Section 3, and change the total sample sizes to 100, 1000, and 5000. Still, the sample are allocated evenly across dosage arms. We present simulation results as Table S4. It can been seen that when $n = 100$, FIL has better performance in terms of MBE and RMSE due to the potential separation issue. As $n = 5000$, the performance of IL and FIL is similar, which is as expected.

3.5 Operating missingness model misspecification pattern

This Section provides outputs when we fit the missingness model omits a predictor with a nonzero effect. In specific, we keep simulation settings the same as in Section 3, while we fit the missingness model with covariate as $z_i = (1, Dose_i, y_i)^\top$. Thus, the nonzero effect covariate X_1 is omitted. We present simulation results as Table S5. It can be seen that both IL and FIL performed best in terms of ED_{50} and E_{max}, while MI has the smallest RMSE when estimating E_0. Due to misspecification modeling on the baseline missingness, IL and FIL are slightly worse than MI, but they still control majority effect due to nonignorable missing.

3.6 Compare MLE and Firth-type modification estimate without missingness

This Section provides outputs when we generated the simulated datasets with no missingness. We compare MLE and Firth-type modification estimate with sample size equal to $n = 150$ to see the potential separation effect. All other settings are the same as in Section 3. We present

Sample Size(N)	Parameter	Type	Estimate	MBE	RMSE	Est.SE	CP	Est.Length
100	$\log(ED_{50})$	CC	1.993	-0.077	1.104	0.939	0.991	3.680
		NRI	2.490	0.475	0.947	0.827	0.923	3.240
		MI	2.206	0.191	0.797	0.884	**0.967**	3.463
		IL	1.938	**-0.022**	0.907	1.205	0.979	4.723
		FIL	2.052	0.037	**0.694**	**0.701**	0.980	**2.749**
	E_{max}	CC	3.930	0.346	0.787	1.032	0.975	4.045
		NRI	4.253	0.669	1.125	1.049	0.990	4.111
		MI	3.755	0.172	**0.813**	0.914	0.981	3.584
		IL	3.561	-0.022	0.875	0.952	**0.959**	3.732
		FIL	3.590	**0.007**	**0.632**	0.898	0.972	**3.520**
	E_0	CC	-2.385	-0.188	0.443	0.932	0.959	3.652
		NRI	-2.611	-0.414	0.583	0.884	1.000	3.465
		MI	-2.039	0.159	0.653	**0.769**	0.930	3.111
		IL	-2.138	0.059	0.411	0.834	0.938	3.270
		FIL	-2.186	**0.011**	**0.390**	0.794	**0.946**	**3.013**
1000	$\log(ED_{50})$	CC	2.690	0.675	2.127	0.326	0.932	1.277
		NRI	3.329	1.314	1.350	0.305	0.016	1.195
		MI	2.589	0.574	0.610	**0.246**	0.339	**0.963**
		IL	2.126	0.112	0.332	0.313	0.945	1.228
		FIL	1.974	**-0.041**	**0.310**	0.312	**0.948**	1.222
	E_{max}	CC	3.948	0.365	0.567	0.426	0.926	1.671
		NRI	4.621	1.037	1.086	**0.319**	0.069	**1.249**
		MI	3.781	0.198	0.588	0.414	0.848	1.623
		IL	3.660	0.076	0.424	0.460	0.962	1.838
		FIL	3.562	**-0.021**	**0.348**	0.425	**0.961**	1.742
	E_0	CC	-2.562	-0.365	0.565	0.420	0.962	1.645
		NRI	-2.985	-0.788	0.846	**0.316**	0.225	**1.239**
		MI	-2.111	0.188	0.433	0.385	0.847	1.342
		IL	-2.009	0.086	0.415	0.408	0.961	1.574
		FIL	-2.277	**-0.080**	**0.290**	0.392	**0.952**	1.499
5000	$\log(ED_{50})$	CC	2.222	0.207	0.241	0.119	0.623	0.468
		NRI	2.791	0.776	0.783	0.104	0.000	0.410
		MI	2.467	0.452	0.462	**0.100**	0.000	**0.394**
		IL	2.039	0.029	0.145	0.130	0.945	0.508
		FIL	2.044	**0.024**	**0.144**	0.128	**0.951**	0.503
	E_{max}	CC	4.083	0.499	0.524	0.165	0.089	0.646
		NRI	4.720	1.137	1.145	0.143	0.000	0.562
		MI	3.816	0.233	0.268	**0.119**	0.525	**0.465**
		IL	3.638	**0.054**	**0.190**	0.165	0.953	0.647
		FIL	3.643	**0.054**	**0.190**	0.165	**0.952**	0.645
	E_0	CC	-2.625	-0.428	0.459	0.162	0.242	0.634
		NRI	-3.089	-0.891	0.903	0.144	0.000	0.563
		MI	-2.309	-0.111	0.184	**0.107**	0.780	0.418
		IL	-2.232	**-0.035**	0.181	0.160	0.951	0.629
		FIL	-2.237	-0.040	**0.180**	0.160	**0.950**	**0.627**

Table S4: Estimates, absolute bias, root mean squared error, estimated standard errors, coverage probabilities and 95% Wald confidence intervals based on 1000 simulations with the same setting as Section 3 across different sample sizes.

Parameter	Type	Estimate	MBE	RMSE	Est.SE	CP	Est.Length
$\log(ED_{50})$	CC	2.061	0.046	0.448	0.425	0.981	1.666
	NRI	2.479	0.465	0.634	0.418	0.803	1.640
	MI	2.268	0.253	0.471	**0.397**	0.925	**1.555**
	IL	1.976	-0.039	0.479	0.442	**0.960**	1.733
	FIL	2.012	**-0.003**	**0.425**	0.404	**0.960**	1.586
E_{max}	CC	3.909	0.325	0.664	0.552	0.907	2.165
	NRI	4.110	0.526	0.744	0.513	0.882	2.012
	MI	3.723	0.140	0.577	**0.463**	0.886	**1.816**
	IL	3.685	**0.102**	0.657	0.540	0.913	2.117
	FIL	3.685	**0.102**	**0.567**	0.522	**0.945**	2.045
E_0	CC	-2.452	-0.254	0.581	0.516	0.975	2.022
	NRI	-2.659	-0.461	0.668	0.490	0.981	1.923
	MI	-2.236	**-0.039**	**0.470**	**0.411**	**0.940**	**1.613**
	IL	-2.240	-0.043	0.598	0.500	0.934	1.960
	FIL	-2.251	-0.054	0.502	0.480	0.938	1.882

Table S5: Estimates, absolute bias, root mean squared error, estimated standard errors, coverage probabilities and 95% Wald confidence intervals based on 1000 simulations with the same setting as Section 3 for missingness model misspecifiaction.

simulation results as Table S6. Both methods converge in all replications. FIL has lower MBE, RMSE, estimated SE, and better coverage probability, indicating that the Firth-type modification indeed controls the estimation bias and variance arising from a small sample setting under the binary Emax model.

Sample Size(N)	Parameter	Type	Estimate	MBE	RMSE	Est.SE	CP	Est.Length
150	$\log(ED_{50})$	MLE	1.957	-0.058	0.676	0.649	0.982	2.546
		Firth	2.058	**0.043**	**0.544**	**0.609**	**0.978**	**2.388**
	E_{max}	MLE	3.926	0.343	1.224	1.036	0.971	4.060
		Firth	3.696	**0.112**	**0.729**	**0.711**	**0.962**	**2.788**
	E_0	MLE	-2.460	-0.262	1.137	0.888	0.969	3.480
		Firth	-2.272	**-0.074**	**0.622**	**0.629**	**0.968**	**2.464**

Table S6: Estimates, absolute bias, root mean squared error, estimated standard errors, coverage probabilities and 95% Wald confidence intervals based on 1000 simulations with full data fitting using MLE and Firth-type modification.

4 Additional result for α estimation

This Section provides outputs of α estimation in the simulation presented in Section 3. Figure 1 to illustrate the distribution of point estimates for α in two proposed methods. The

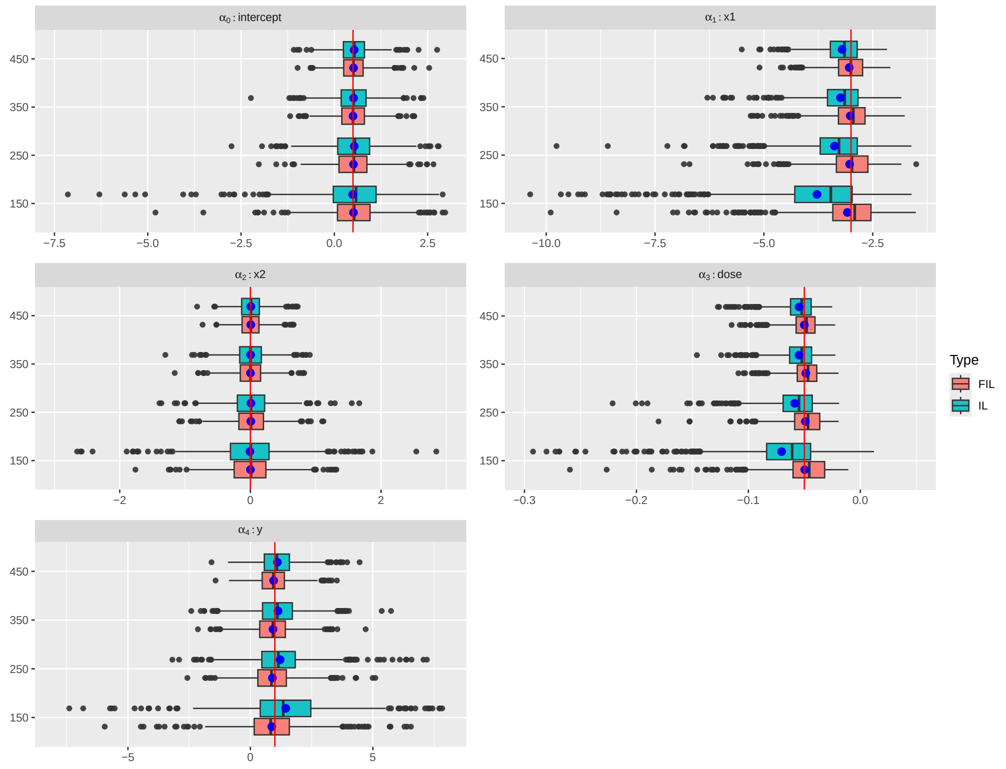

Figure 1: Boxplots comparing the distribution of point estimates of alpha, based on 1000 replications and missing rate approximately 15%.

true value of α is indicated by a vertical line on the boxplot, and the mean estimates are shown as blue dots. The reuslts show that under the assumed missingness model containing all nonzero missingness predictors, α_4, the coefficient of y, is identified. The other coefficients are estimated properly as sample sizes increase. FIL still outperforms IL in terms of variance and bias when the sample size is small.
